# Sequence alignment by passing messages

**DOI:** 10.1186/1471-2164-15-S1-S14

**Published:** 2014-01-24

**Authors:** Byung-Jun Yoon

**Affiliations:** Department of Electrical and Computer Engineering, Texas A&M University, College Station, TX 77843-3128 USA

## Abstract

**Background:**

Sequence alignment has become an indispensable tool in modern molecular biology research, and probabilistic sequence alignment models have been shown to provide an effective framework for building accurate sequence alignment tools. One such example is the pair hidden Markov model (pair-HMM), which has been especially popular in comparative sequence analysis for several reasons, including their effectiveness in modeling and detecting sequence homology, model simplicity, and the existence of efficient algorithms for applying the model to sequence alignment problems. However, despite these advantages, pair-HMMs also have a number of practical limitations that may degrade their alignment performance or render them unsuitable for certain alignment tasks.

**Results:**

In this work, we propose a novel scheme for comparing and aligning biological sequences that can effectively address the shortcomings of the traditional pair-HMMs. The proposed scheme is based on a simple message-passing approach, where messages are exchanged between neighboring symbol pairs that may be potentially aligned in the optimal sequence alignment. The message-passing process yields probabilistic symbol alignment confidence scores, which may be used for predicting the optimal alignment that maximizes the expected number of correctly aligned symbol pairs.

**Conclusions:**

Extensive performance evaluation on protein alignment benchmark datasets shows that the proposed message-passing scheme clearly outperforms the traditional pair-HMM-based approach, in terms of both alignment accuracy and computational efficiency. Furthermore, the proposed scheme is numerically robust and amenable to massive parallelization.

## Background

Sequence alignment has become an indispensable tool in modern molecular biology research, as it provides an effective and intuitive way of comparing and analyzing biological sequences. Given a set of biological sequences, the primary objective of sequence alignment is to predict the best overall mapping between the sequences, which accurately aligns the homologous regions that are embedded in them. This provides an effective means for detecting conserved sequence regions with potentially important functional roles. The concept of sequence alignment has had diverse applications in biomedical research [1–7], which include homology search, function and structure prediction of biomolecules, phylogenetic analysis, and detecting sequence motifs, among others.

Typically, sequence alignment is carried out by formulating and solving an optimization problem - either implicitly or explicitly - where the goal is to maximize an objective function that measures the overall quality of the sequence alignment. For example, one simple way of aligning a sequence pair would be to score each potential alignment by assigning a "substitution score" to every aligned symbol pair and penalty scores for gaps and then find the optimal alignment that maximizes the overall score through dynamic programming [1]. In the past, various *ad hoc* scoring schemes have been proposed to obtain intuitive and biologically meaningful sequence alignment results. As an alternative to heuristic scoring schemes, there have been also research efforts to develop probabilistic models for sequence alignment that can be used to evaluate and compare potential alignments and to estimate the symbol-to-symbol alignment probabilities.

Examples of such probabilistic schemes include the pair hidden Markov models (pair-HMMs) [1] and the partition function based scheme [8]. Given two biological sequences, these methods can be used to estimate the posterior symbol alignment probability for each symbol pair that may be aligned in the final sequence alignment. Based on the estimated probabilities, we can predict the optimal sequence alignment that contains the largest expected number of correctly aligned symbol pairs, rather than an alignment that maximizes an *ad hoc* score. This is typically referred to as the maximum expected accuracy (MEA) alignment [9–11], and as before, it can be also found through dynamic programming.

Among a number of probabilistic sequence alignment models, pair-HMMs have been especially popular, and they have been widely adopted by many multiple sequence alignment (MSA) algorithms, including ProbCons [9] and PicXAA [10]. Despite the simplicity of the model, pair-HMMs have been shown to be very effective in modeling sequence homology, as reflected in the well-rounded overall performance of various MSA algorithms that utilize the symbol alignment probabilities estimated by pair-HMMs. Furthermore, these probabilities can be estimated in a relatively efficient manner, making the pair-HMMs an attractive choice for various sequence alignment problems. However, pair-HMMs also have a number of shortcomings, which may negatively affect their alignment performance or make them impractical for certain alignment tasks.

In this paper, we propose a novel scheme for comparing and aligning biological sequences that can effectively address the limitations of pair-HMMs. The proposed scheme computes probabilistic symbol alignment confidence scores based on a simple and computationally efficient message-passing approach. As we will demonstrate in this paper, this message-passing scheme has a number of important advantages over the traditional pair-HMMs and it clearly outperforms pair-HMMs in terms of both speed and accuracy on protein alignment benchmark datasets.

## Methods

### A brief overview of pair hidden Markov models

**Figure 1:**
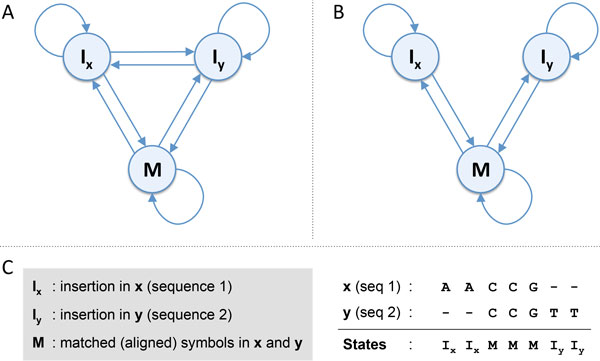
**Pair hidden Markov models**. (A) The state transition diagram of a widely used pair-HMM. (B) An alternative pair-HMM implementation that does not allow transitions between the two insertion states *I*_*x*_ and *I*_*y*_. (C) An example of a sequence pair (**x**, **y**) that is generated by a pair-HMM.

The pair-HMM [1, 12] is a generative sequence model that can simultaneously generate a *pair* of aligned symbol sequences. This is different from the traditional HMMs, which generate only a single symbol sequence at a time [13]. Figure 1 shows two examples of pair-HMMs that are widely used in biological sequence analysis. As shown in Figure 1, a typical pair-HMM consists of three hidden states I_x_, I_y_, and M, which are used to model insertions in sequence **x**, insertions in sequence **y**, and matched (i.e., aligned) symbols in both sequences, respectively. The pair-HMM generates an aligned sequence pair (**x**, **y**) by making transitions between the hidden states according to the specified state transition probabilities. At state I_x_, the model emits a symbol only to sequence **x**, while at I_y_, a symbol is emitted only to sequence **y**. On the other hand, at state M, the model emits a pair of aligned symbols, where one symbol is added to **x** and the other symbol is added to **y**. Figure 1(C) gives an example of a sequence pair (**x**, **y**) that is generated by a pair-HMM. In this example, the underlying hidden state sequence that gives rise to the two sequences **x** = AACCG and **y** = CCGTT is I_x_I_x_MMMI_y_I_y_. This indicates that the first two symbols (i.e., AA) in **x** and the last two symbols in **y** (i.e., TT) are "insertions," which do not have any matching counterpart in the other sequence, while the last three symbols in **x** and the first three symbols in **y** (i.e., CCG in both sequences) are jointly generated by the pair-HMM, hence closely match each other. As we can see from this example, we can unambiguously identify the alignment of a given sequence pair (**x**, **y**), once the underlying hidden state sequence yielding the sequence pair is known. Of course, the hidden state sequence is generally not known, but there exist efficient algorithms that can be used for its prediction. For example, we can use the Viterbi algorithm [14] to predict the optimal hidden state sequence that maximizes the observation probability of the sequence pair (**x**, **y**). Alternatively, we can also predict the state sequence that maximizes the expected number of correct states, by first estimating the alignment probabilities between the symbols in **x** and **y** through the forward and backward procedures [13] and then applying the Needleman-Wunsch algorithm [15]. This will lead to the MEA alignment between the two sequences **x** and **y**.

### Limitations of pair-HMMs

Although the hidden state sequence of a pair-HMM unambiguously points to a specific sequence alignment, this is not necessarily true the other way around. In fact, several different state sequences can lead to the same sequence alignment, hence we may not always be able to unambiguously determine the underlying state sequence for a given pairwise sequence alignment. For example, let us consider two sequences **x** = AAACGG and **y** = AAATTA. Suppose the "true" alignment aligns only the first three symbols (i.e., AAA) of **x** and **y**, hence the last three symbols in the respective sequences are regarded as insertions that do not have any matching counterpart in the other sequence. This is illustrated below, where the solid lines correspond to the aligned symbols:1

For the pair-HMM shown in Figure 1(A), any hidden state sequence  such that *s*_1_ = *s*_2_ = *s*_3_ = M and  is a permutation of I_x_ I_x_ I_x_ I_y_ I_y_ I_y_ would lead to the sequence alignment shown in (1). When using this pair-HMM for predicting the optimal alignment of a sequence pair with the largest probability, this ambiguity may lead to performance degradation as these potential state sequences compete against each other. For this reason, it is generally more desirable to estimate the symbol alignment probabilities via the pair-HMM by considering all potential alignments and state sequences and use the estimated probabilities to find the MEA alignment that is expected to have the maximum number of correctly aligned symbols [9–11]. However, the aforementioned ambiguity also negatively affects the quality of the estimated symbol alignment probabilities, which is especially noticeable for sequence pairs with low percentage identity. In some cases, the alternative pair-HMM shown in Figure 1(B) is used to avoid such ambiguity. This alternative pair-HMM blocks transitions between the insertion states I_x_ and I_y_, thereby prohibiting the model from inserting unaligned symbols to both sequences. For example, the alignment shown in (1) would not be allowed based on this alternative pair-HMM. However, due to this restriction, the pair-HMM in Figure 1(B) has a relatively stronger tendency to align unrelated sequence regions by treating them as mutations. This may again negatively affect the quality of the symbol alignment probabilities estimated based on the pair-HMM.

Another potential drawback of pair-HMMs is that the associated algorithms (i.e., the Viterbi, forward, and backward algorithms) can become numerically unstable for long sequences. Application of pair-HMMs to biological sequence analysis involves computing extremely small probabilities, which decrease exponentially with the sequence length. For example, based on the pair-HMM that was used in [9], the observation probability (i.e., the probability that the HMM may generate a given sequence pair) of a protein pair is typically in the order of 10^-230^ for proteins of length 80, 10^-280^ for proteins of length 100, and 10^-320^ for proteins of length 120. As a result, pair-HMM algorithms are prone to underflow errors, unless they are carefully implemented to keep them numerically robust. So far, a number of schemes have been proposed to address this issue, such as using log transformations of the probabilities or normalizing the probabilities to keep them within a reasonable numerical range, and have been shown to work well for relatively long sequences [1]. However, log transformations can make the forward and backward algorithms considerably slower, and the normalization approach can still lead to underflow errors as the sequences get longer.

One further disadvantage of pair-HMMs is that the algorithms that are used with the model cannot be easily parallelized. Although the Viterbi, forward, and backward algorithms for pair-HMMs are relatively efficient, they are still computationally expensive to be used with very long sequences. Moreover, as the algorithms are not amenable to massive parallelization, this makes the pair-HMMs not suitable for large-scale sequence analysis tasks, such as the whole genome alignment, despite their superior performance compared to other heuristic methods.

### A message-passing scheme for estimating symbol alignment confidence scores

Here, we propose a novel method for aligning biological sequences that can effectively address the aforementioned shortcomings of pair-HMMs. The proposed method is based on a message-passing scheme, where messages are iteratively exchanged between neighboring symbol pairs to estimate the level of confidence for potential pairwise symbol alignments. The main underlying motivation is to develop an "analytical" method that can *directly* estimate the symbol alignment probabilities, without specifically modeling symbol insertions and deletions. This stands in contrast to the pair-HMM approach, which is essentially based on a "generative" sequence model that tries to explicitly model symbol insertions/deletions, in addition to symbol alignments. As discussed before, modeling symbol insertions in pair-HMMs can lead to subtle issues with potentially negative effects, and considering that our ultimate goal lies in finding an accurate sequence alignment through effective estimation of the symbol alignment probabilities, a method that can directly estimate these probabilities without explicitly modeling insertions/deletions would be desirable.

**Figure 2:**
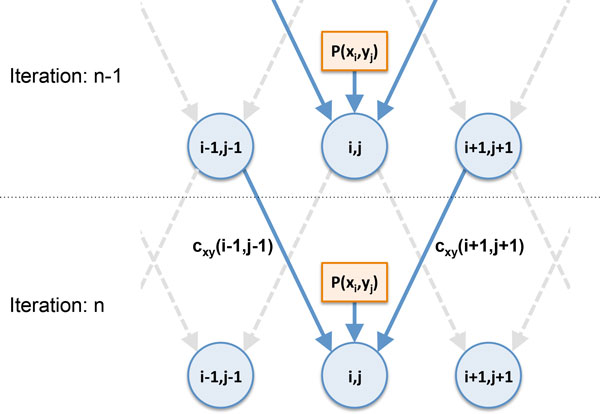
**Illustration of the proposed message-passing scheme**. At iteration *n*, the alignment confidence score *c*_**xy**_(*i*, *j*) of the symbol pair (*x*_*i*_, *y*_*j*_) is updated based on the messages received from its neighbors (*x*_*i*-1_, *y*_*j*-1_) and (*x*_*i*+1_, *y*_*j*+1_) and the joint occurrence probability *P*(*x*_*i*_, *y*_*j*_) of the symbols *x*_*i*_ and *y*_*j*_. Solid lines indicate the messages that are used to update *c*_**xy**_(*i*, *j*), while the dashed lines correspond to messages that are used to update the alignment confidence scores of other symbol pairs.

Suppose **x** = *x*_1_*x*_2_ ⋯ *x*_*L*_ and **y** = *y*_1_*y*_2_ ⋯ *y*_*M*_ are the two sequences to be aligned. We define *c*_**xy**_ (*i*, *j*) as the *symbol alignment confidence score* between *x*_*i*_ (the *i*-th symbol in **x**) and *y*_*j*_ (the *j*-th symbol in **y**). The score *c*_**xy**_ (*i*, *j*) provides a quantitative measure of confidence as to whether *x*_*i*_ and *y*_*j*_ should be aligned to each other or not, and we assume *c*_**xy**_ (*i*, *j*) ∝ *P*(*x*_*i*_*~ y*_*j*_|**x**, **y**), where *P* (*x*_*i*_ ~ *y*_*j*_|**x**, **y**) is the posterior symbol alignment probability between *x*_*i*_ and *y*_*j*_ given the sequences **x** and **y**. We estimate the alignment confidence score by iteratively passing messages between neighboring symbol pairs, where each symbol pair (*x*_*i*_, *y*_*j*_) corresponds to a potential symbol alignment in the true (unknown) sequence alignment between **x** and **y**. For example, during the estimation process, the symbol pair (*x*_*i*_, *y*_*j*_) will exchange messages with its two neighbors (*x*_*i*-1_, *y*_*j*-1_) and (*x*_*i*+1_, *y*_*j*+1_), and similarly, the pair (*x*_*i*+1_, *y*_*j*+1_) will exchange messages with (*x*_*i*_, *y*_*j*_) and (*x*_*i*+2_, *y*_*j*+2_). The message-passing process is illustrated in Figure 2, where the solid lines indicate the messages that are used to update the alignment confidence score *c*_**xy**_(*i*, *j*) of the symbol pair (*x*_*i*_, *y*_*j*_). The dashed lines correspond to messages that are used to update the confidence scores of other symbol pairs.

The pseudocode of the proposed message-passing algorithm is as follows:

   STEP-1 Initialize*c*_**xy**_ (*i*, *j*).

   STEP-2 Update the alignment confidence score:   

   STEP-3 Normalize*c*_**xy**_(*i*, *j*).

   STEP-4 If*c*_**xy**_(*i*, *j*) has converged, then terminate the algorithm.

                  Otherwise, go to STEP-2.

In STEP-1, we first initialize the alignment confidence score *c*_**xy**_(*i*, *j*), where we can simply use random initialization. If a preliminary sequence alignment of **x** and **y** is available (e.g., obtained from a simple heuristic method), we can also initialize the score based on this alignment such that *c*_**xy**_(*i*, *j*) = 1 if *x*_*i*_ and *y*_*j*_ are aligned, and *c*_**xy**_(*i*, *j*) = 0 otherwise. Next, in STEP-2, the alignment confidence score *c*_**xy**_(*i*, *j*) of the symbol pair (*x*_*i*_, *y*_*j*_) is updated based on the scores of its two neighbors (*x*_*i*-1_, *y*_*j*-1_) and (*x*_*i*+1_, *y*_*j*+1_). Note that the score is set to *c*_**xy**_(*i*, *j*) = 0 if *i* ∉ {1, *⋯* , *L*} or *j* ∉ {1, *⋯* , *M*}. *P* (*x*_*i*_, *y*_*j*_) is the joint occurrence probability of the symbol pair (*x*_*i*_, *y*_*j*_), which is essentially equivalent to the joint emission probability of an aligned symbol pair (*x*_*i*_, *y*_*j*_) at the match state M of a pair-HMM. It should be noted that this probability *P* (*x*_*i*_, *y*_*j*_) is not location-dependent and is simply determined by the symbols *x*_*i*_ and *y*_*j*_. The weight parameter *λ* ∈ [0, 1] is used to balance the contribution from the neighbors and that from the joint probability of (*x*_*i*_, *y*_*j*_) in estimating the alignment confidence score. A large *λ* gives more weight to the "messages" received from the neighbors in estimating the scores, which tends to penalize gaps more heavily, and it generally leads to longer aligned regions with fewer gaps. On the contrary, a small *λ* gives more weight to the joint symbol occurrence probability *P* (*x*_*i*_, *y*_*j*_) while giving less weight to the messages received from the neighbors, which tends to be more lenient to gaps. Once the symbol alignment confidence score *c*_**xy**_(*i*, *j*) is updated for all *i* = 1, *⋯* , *L* and *j* = 1, ⋯ , *M*, we normalize the scores to keep them within a proper numerical range, as shown in STEP-3. For example, a simple way would be to divide the score matrix **C** = [(*c*_**xy**_(*i*, *j*)] by its matrix norm to normalize the confidence scores. After normalization, the updated scores are compared to the scores in the last iteration, and the algorithm terminates if the specified convergence criterion has been met. Otherwise, the algorithm goes back to STEP-2 and repeats the message-passing process.

## Results and Discussion

### Dataset and experimental set-up

In order to evaluate the performance of the proposed message-passing scheme, we carried out pairwise sequence alignment experiments based on the BAliBASE 3.0 protein alignment benchmark [16]. BAliBASE is arguably the most widely used benchmark for multiple sequence alignment, and it has been utilized by most multiple sequence alignment algorithms for assessing their performance. The benchmark consists of five reference sets, where Reference 1 consists of two subsets: V1 and V2. Each reference set consists of multiple sequence alignments that satisfy specific criteria, such that different reference sets can be used to test the performance of multiple sequence alignment algorithms under different conditions. For example, each alignment in Reference 2 consists of sequences that share reasonably high identity (> 40%) and "orphan sequences" that share little identity (< 20%) to other sequences in the alignment. Reference sets 4 and 5 are constructed such that every sequence has at least one other sequence in the same alignment whose identity exceeds 20%. Sequences in Reference 4 and Reference 5 may contain large N/C-terminal extensions or internal insertions, respectively. Further details of the BAliBASE 3.0 benchmark can be found in [16].

For every sequence family in BAliBASE 3.0, we performed pairwise sequence alignment for all possible sequence pairs in the given family. The pairwise alignment was performed in the following manner. First, we estimated the probabilistic symbol alignment confidence score using the proposed message-passing scheme. In our experiments, we used three different values of *λ* (= 0.25, 0.5, and 0.75) to investigate the effect of *λ* on the overall sequence alignment performance. For the joint symbol occurrence probability *P* (*x*_*i*_, *y*_*j*_), we used the joint emission probability (at state M) of the pair-HMM that was used in [9]. At the end of each iteration, we normalized the alignment confidence score by dividing the confidence score matrix **C** by the matrix 2-norm: **C***←***C***/||***C***||*_2_. We terminated the message-passing process if , where *c*_**xy**_(*i*, *j*) is the current score and  is the score obtained in the previous iteration. Once the scores converged, based on our assumption that *c*_**xy**_ (*i*, *j*) ∝ *P* (*x*_*i*_ ~ *y*_*j*_*|***x**, **y**), we used the confidence score *c*_**xy**_ (*i*, *j*) to find the MEA alignment through dynamic programming. The predicted alignment was compared to the benchmark alignment in BAliBASE 3.0 to compute the sensitivity  and the positive predictive value , where *TP* is the number of correctly aligned symbol pairs, *FP* is the number of incorrectly aligned pairs, and *FN* is the number of symbol pairs that are aligned in the benchmark alignment but not aligned in the predicted alignment. For comparison, we repeated similar experiments using the pair-HMM with the same set of parameters as the one used in [9].

### Performance of the proposed message-passing scheme

Table 1 summarizes the pairwise sequence alignment performance of the proposed message-passing scheme and the traditional pair-HMM approach. Each row shows the evaluation results on each of the six reference sets (i.e., RV11, RV12, RV20, RV30, RV40, RV50) in BAliBASE 3.0. For each reference set, we estimated the average SN, PPV, and CPU time (for estimating the alignment scores/probabilities) of different alignment schemes based on all possible pairwise sequence alignments: 943 alignments for the reference set RV11, 2,335 alignments for RV12, 50,062 alignments for RV20, 76,370 alignments for RV30, 23,445 alignments for RV40, and 7,538 alignments for RV50. All experiments were performed using Matlab on a MacPro workstation with two 2.8 GHz Quad-Core Intel Xeon processors and 32GB memory.

**Table 1:** Pairwise sequence alignment performance evaluated on the BAliBASE 3.0 benchmark.

Ref	Pair-HMM						Message-Passing					
				***λ*** **= 0.25**			***λ*** **= 0.50**			***λ*** **= 0.75**		
	**SN**	**PPV**	**CPU**	**SN**	**PPV**	**CPU**	**SN**	**PPV**	**CPU**	**SN**	**PPV**	**CPU**
RV11	0.048	0.106	1.934	0.123	0.149	**0.769**	0.155	0.175	1.465	**0.198**	**0.209**	3.675
RV12	0.213	0.414	2.707	0.399	0.468	**1.146**	0.475	0.523	2.145	**0.569**	**0.595**	5.200
RV20	0.276	0.476	2.725	0.504	0.568	**1.186**	0.570	0.613	2.251	**0.643**	**0.665**	5.531
RV30	0.168	0.300	2.656	0.324	0.369	**1.143**	0.372	0.402	2.160	**0.426**	**0.441**	5.432
RV40	0.153	0.271	4.084	0.250	0.284	**1.760**	0.300	0.323	3.234	**0.361**	**0.373**	7.970
RV50	0.140	0.254	4.969	0.248	0.278	**2.102**	0.294	0.312	3.967	**0.348**	**0.353**	9.856

The average sensitivity (SN), positive predictive value (PPV), and CPU time (seconds) on different reference sets are shown for each sequence alignment scheme. All experiments were performed in Matlab on a MacPro workstation with 2 × 2.8 GHz Quad-Core Intel Xeon processors and 32GB memory.

From Table 1, we can clearly see that the proposed message-passing scheme significantly outperforms the pair-HMM approach in terms of SN and PPV, for all three values of *λ*. For example, the message-passing scheme achieved up to 0.23 higher SN and 0.09 higher PPV for *λ* = 0.25, and up to 0.37 higher SN and 0.19 higher PPV for *λ* = 0.75. Our experiments showed that a larger *λ* tends to yield more accurate alignments, while a smaller *λ* tends to make the algorithm converge faster, hence computationally more efficient. For example, when the weight parameter was set to *λ* = 0.25, the message-passing scheme was around 2.3 ~ 2.5 times faster than the pair-HMM, while still yielding much more accurate alignments.

**Figure 3:**
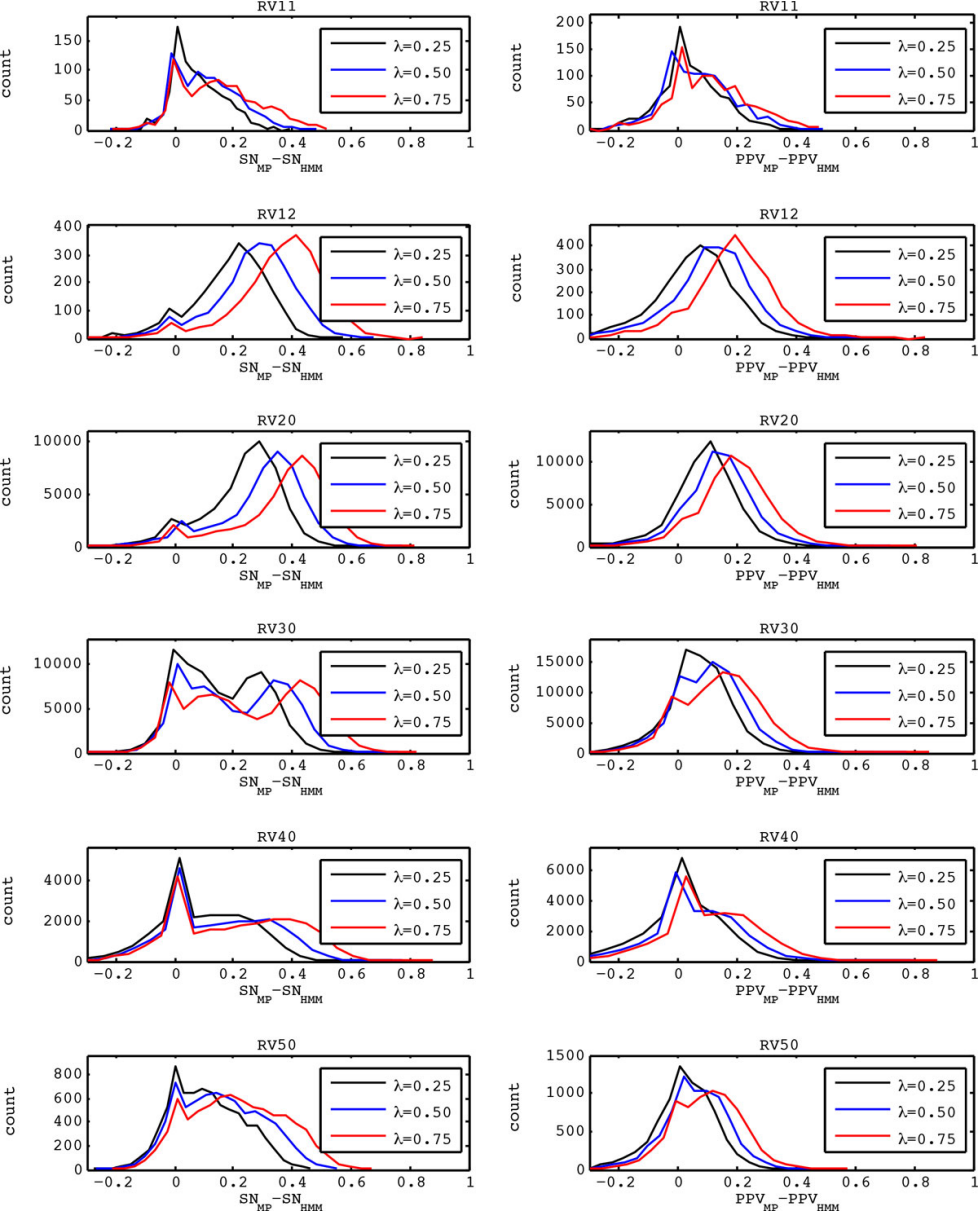
**Performance comparison between the proposed message-passing scheme and the traditional pair-HMM approach**. The plots in the left column show the distributions of the sensitivity difference *SN*_*MP*_ *-SN*_*HMM*_ between the message-passing scheme and the pair-HMM-based approach. In the right column, the distributions of the difference between the positive predictive values *PPV*_*MP*_ *- PPV*_*HMM*_ of the two schemes are shown. Each row shows the evaluation results obtained from each of the six reference sets in BAliBASE 3.0.

The results in Table 1 demonstrate that, on average, the proposed message-passing scheme considerably improves the quality of sequence alignment over the traditional pair-HMM approach. In order to see whether the proposed scheme also leads to a consistent improvement for most sequence pairs, we calculated the difference between *SN*_*MP*_ (the sensitivity of the message-passing scheme) and *SN*_*HMM*_ (the sensitivity of the pair-HMM-based approach) for every pairwise sequence alignment that we have performed in our experiments. Similarly, we calculated the difference between *PPV*_*MP*_ (the PPV of the message-passing scheme) and *PPV*_*HMM*_ (the sensitivity of the pair-HMM approach) for all sequence pairs in BAliBASE 3.0. Figure 3 shows the distributions of *SN*_*MP*_*- SN*_*HMM*_ and *PPV*_*MP*_*- PPV*_*HMM*_ for all sequence pairs. To avoid any bias from unsuccessful alignments, sequence pairs for which neither method yielded an alignment with at least one correct symbol alignment were excluded. The plots in the left column of Figure 3 show the distributions of *SN*_*MP*_*- SN*_*HMM*_, and those in the right column show the distributions of *PPV*_*MP*_*- PPV*_*HMM*_. The results obtained from the same reference set are shown in the same row, where the first row shows the results on RV11 and the last row shows the results on RV50. As we can see in Figure 3, every single distribution shown in the figure has a much larger probability mass in the right-half plane, which clearly demonstrates that the proposed message-passing scheme consistently outperforms the pair-HMM-based approach for most (though not all) sequence pairs. In many cases, the improvements in SN and PPV were quite significant (0.4 ~ 0.8), which shows that the proposed scheme can often find an accurate sequence alignment even when the pair-HMM has difficulty aligning the sequences.

## Conclusions

In this paper, we proposed a novel method for sequence alignment based on an efficient message-passing approach. Given two biological sequences, the proposed method estimates the symbol alignment confidence scores for all possible symbol pairs. These scores are iteratively computed by exchanging messages between neighboring symbol pairs, where empirical evidence shows that these scores quickly converge within several iterations. The proposed message-passing scheme effectively addresses a number of limitations of the traditional pair-HMM-based approach, and extensive performance assessment based on BAliBASE 3.0 shows that the proposed scheme consistently outperforms the pair-HMM approach, both in terms of alignment accuracy and computational efficiency. Considering that pair-HMMs have been widely adopted by many modern multiple sequence alignment algorithms [9–11], the proposed scheme has potentials to further improve the current state-of-the-art. Furthermore, the proposed scheme is numerically stable even for extremely long sequences. Unlike the pair-HMM approach, there is no global measure or quantity (such as the observation probability *P* (**x**, **y**) of the entire sequence pair) to be estimated, and the exchanged messages (i.e., symbol alignment confidence scores) are normalized after each iteration, which ensures that they lie within a reasonable numerical range. Finally, the simple iterative estimation process - in which the neighboring symbol pairs only exchange "local" messages - makes the proposed message passing scheme amenable to massive parallelization through the utilization of modern GPU (graphics processing unit) architecture. These characteristics open up the possibility of applying the proposed message-passing scheme to accurate probabilistic alignment of genome-scale sequences, which has not been possible using traditional pair-HMMs.

Finally, it is worth noting that the formula that is used to update *c*_**xy**_(*i*, *j*) in the proposed message-passing algorithm bears conceptual similarity to the eigenvalue equation used by the network alignment algorithm called IsoRank [17] for estimating the functional similarity between proteins across different protein-protein interaction (PPI) networks. As demonstrated in [18, 19], techniques that were originally developed for sequence alignment may also have potentials to improve network alignment methods. Conversely, techniques used in network alignment may also lead to better sequence alignment methods. For example, the scoring scheme used by IsoRank can be viewed as a random walk [20], and it was shown that the use of a different random walk scheme can lead to more accurate network alignment results [19]. Similarly, it may be possible to modify the update formula for *c*_**xy**_(*i*, *j*) to further improve the performance of the proposed message-passing scheme, and we are currently in the process of investigating several different implementations.
